# Noisy splicing, more than expression regulation, explains why some exons are subject to nonsense-mediated mRNA decay

**DOI:** 10.1186/1741-7007-7-23

**Published:** 2009-05-14

**Authors:** Zhenguo Zhang, Dedong Xin, Ping Wang, Li Zhou, Landian Hu, Xiangyin Kong, Laurence D Hurst

**Affiliations:** 1 Institute of Health Sciences, Shanghai Institutes for Biological Sciences (SIBS), Chinese Academy of Sciences (CAS) & Shanghai Jiao Tong University School of Medicine (SJTUSM), Shanghai, PR China; 2 Graduate School of the Chinese Academy of Sciences, Shanghai, PR China; 3 State Key Laboratory of Medical Genomics, Ruijin Hospital, Shanghai Jiaotong University, 197 Rui Jin Road II, Shanghai, PR China; 4 Department of Biology and Biochemistry, University of Bath, Bath, UK

## Abstract

**Background:**

Nonsense-mediated decay is a mechanism that degrades mRNAs with a premature termination codon. That some exons have premature termination codons at fixation is paradoxical: why make a transcript if it is only to be destroyed? One model supposes that splicing is inherently noisy and spurious transcripts are common. The evolution of a premature termination codon in a regularly made unwanted transcript can be a means to prevent costly translation. Alternatively, nonsense-mediated decay can be regulated under certain conditions so the presence of a premature termination codon can be a means to up-regulate transcripts needed when nonsense-mediated decay is suppressed.

**Results:**

To resolve this issue we examined the properties of putative nonsense-mediated decay targets in humans and mice. We started with a well-annotated set of protein coding genes and found that 2 to 4% of genes are probably subject to nonsense-mediated decay, and that the premature termination codon reflects neither rare mutations nor sequencing artefacts. Several lines of evidence suggested that the noisy splicing model has considerable relevance: 1) exons that are uniquely found in nonsense-mediated decay transcripts (nonsense-mediated decay-specific exons) tend to be newly created; 2) have low-inclusion level; 3) tend not to be a multiple of three long; 4) belong to genes with multiple splice isoforms more often than expected; and 5) these genes are not obviously enriched for any functional class nor conserved as nonsense-mediated decay candidates in other species. However, nonsense-mediated decay-specific exons for which distant orthologous exons can be found tend to have been under purifying selection, consistent with the regulation model.

**Conclusion:**

We conclude that for recently evolved exons the noisy splicing model is the better explanation of their properties, while for ancient exons the nonsense-mediated decay regulated gene expression is a viable explanation.

## Background

**Figure 1:**
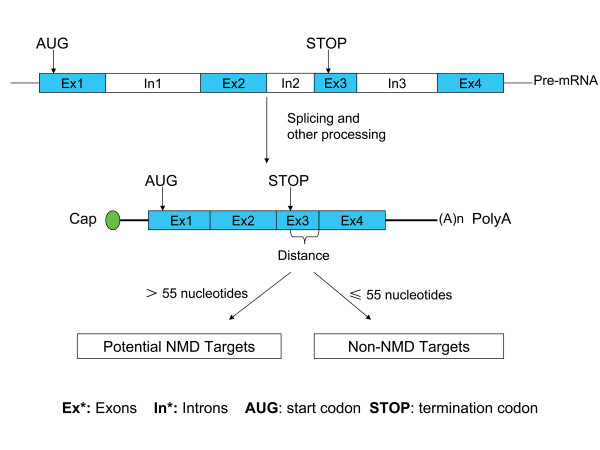
**The mammalian nonsense-mediated mRNA decay (NMD) rule**. Stop codons located > 50 to 55 nucleotides upstream of the 3' most splice-generated exon-exon junction usually trigger NMD in mammals [16]. Mammalian genes are transcribed from the genome, which produces the precursor of mRNA (pre-mRNA). Pre-mRNA still contains exons (Ex) and introns (In), and is subject to processes including capping, polyadenylation and splicing. The splicing step removes the introns from pre-mRNA and ligates the exons. The spliced mRNA then undergoes the first round of translation during export [17]. If the distance from the normal stop codon to the exon-exon junction closest to the 3'-end (labelled 'Distance' in the figure) is > 55 nucleotides, in most cases the mRNA will be degraded by the NMD pathway – we refer to these transcripts as putative NMD targets. mRNAs for which the distance from the normal stop codon to the exon-exon junction closest to the 3'-end is < 50 to 55 nucleotides are free of NMD decay. These mRNAs and ones with stop codons in the last exon are classified as putative non-NMD targets.

Nonsense-mediated mRNA decay (NMD) is a mechanism for rapid degradation of mRNA transcripts with premature stop/termination codons (PTCs) [1-7]. Quite how a cell knows that a stop codon is 'premature' is taxonomically variable [2,4,8-12]. In *Saccharomyces cerevisiae *and *Drosophila melanogaster *a termination codon is determined as a PTC when positioned too far upstream of a poly(A) tail [13-15]. In mammals, the species we consider here, the recognition of premature termination codons generally depends on the distance between nonsense codons and the exon-exon junction closest to the 3' end. When this distance is > 50 to 55 nucleotides, NMD is triggered and the mRNA is degraded [1,16]. This is known as the NMD 55-nt rule (Figure 1) [16,17].

Note that the 55-nt rule will not capture all NMD subject transcripts. Singh *et al*. found that an artificial 3' untranslated region (UTR) of > 420 nucleotides can stimulate NMD independent of the 55-nt rule [18]. Upstream open reading frames (uORFs) can also trigger NMD in a size-dependent manner [19]. Furthermore, cytoplasmic poly(A)-binding protein (PABP) inhibits the interaction between eRF3 and Upf1 *in vitro *and prevents NMD in cells when positioned in proximity to the termination codon [15,18-20]. Based on these findings, a unified model is proposed that the distinction between translation termination at PTCs and at 'normal' termination codons relies on the physical distance between the terminating ribosome and PABP [21]. Nonetheless, the 55-nt rule is the best defined and unlikely to greatly mislead.

That NMD is an important mechanism is witnessed by the fact that the malfunction of NMD results in serious consequences. In mice, loss of *Rent1 *(*UPF1*), a key factor of NMD, leads to death at an early embryonic developmental stage [22]. Moreover, approximately one-third of inherited genetic disorders and many forms of cancers are associated with mutant genes containing PTCs [23-26].

The fact of NMD raises numerous questions. How many genes have transcripts subject to NMD? More particularly, beyond the occasional rare allele, why do many genes have PTCs? Their existence is at first sight paradoxical: why do cells make a transcript only for it to be degraded? Given that transcripts are degraded to a certain extent, might NMD genes be subject to weaker purifying selection and might their relative freedom to explore sequence space ensure that they are hot-spots for further adaptive changes? Here we define a set of RefSeq genes that are likely to be subject to NMD so as to investigate the above issues.

As regards the central paradox of NMD, two hypotheses are prominent [27,28]. First, one can suppose that splicing is an inherently error-prone process regularly throwing up the same unwanted transcripts [29,30]. This being so, NMD can degrade these non-functional transcripts avoiding costly-to-make, potentially toxic, proteins. We consider this the noisy or spurious transcript model. Second, the NMD machinery need not always be operative and can be regulated. It can, for example, be suppressed under nutrient-limiting conditions [10]. Similarly, both levels of *RNPS1 *(an exon junction component) and hypoxia can modulate NMD intensity [31,32]. NMD could then be a mechanism to permit up-regulation of specific transcripts on suppression of NMD. This we dub the regulation model.

These two models make numerous predictions about the properties and evolution of NMD target genes and exons. The spurious transcript model predicts that NMD genes should have more than one splice isoform. The regulation model need not predict the same. The noisy model would predict that spurious exons that are not multiples of three (and hence induce frameshifts) should be more likely to provide the selective conditions favouring an in-frame PTC to prevent translation [29]. The noisy model additionally predicts that the NMD-inducing exons should be in rare transcripts and more common in recently evolved exons. By contrast, the regulation model predicts that the NMD-specific exons should be under purifying selection and that the NMD regulation of orthologous genes be conserved in relatively distant species.

Prior evidence can be given to support both models. As regards the regulation model, up-regulation of enzymes associated with amino acid biosynthesis on NMD inactivation, for example, has been related to a feedback circuit coupling low amino acid levels with inactivation of NMD and hence increased translation rates of mRNAs associated with amino acid biosynthesis [10]. More interestingly, it has been reported that NMD is coupled with alternative splicing to regulate a variety of genes [28]. For instance, ribosomal mRNAs (for example *rpL3*, *rpL12*) [33,34] and splicing-related factors (for example, *SC35*, *PTB*) [35-37] are auto-regulated by NMD and alternative splicing. Related to this finding, tens of conserved stop-containing exons whose inclusion renders the transcript sensitive to NMD are found in mice, and these exons are unusually frequent in genes that encode splicing activators (such as serine/arginine-rich proteins) and are unexpectedly enriched in the so-called 'ultraconserved' elements in the mammalian lineage [38].

Other evidence supports the noisy splice model. Several studies have made efforts to identify and study the naturally occurring transcripts regulated by NMD [9-11,39-42]. By aligning expressed sequence tags (ESTs) on genomic regions to infer splicing isoforms, about 35% of alternative splicing events are predicted to have the potential to produce PTC-containing transcripts [40,42]. Using a similar method, Baek and Green also found that about 20% of conserved alternative splicing events produced PTCs [43]. Based on the full-length transcripts, Xing and Lee found that 11% of alternatively spliced isoforms contained PTCs [44]. Using an alternative splicing microarray platform Pan and co-workers found that most of the PTC-containing transcripts were low in abundance across examined tissues, and this low abundance was independent of NMD function [41], arguing against the regulation function of NMD. Furthermore, comparative analysis shows that NMD-inducing alternative splicing events are not conserved between humans and mice [41] suggesting noise above regulation, as does the finding that comparison of experimentally identified *S. cerevisiae*, *D. melanogaster *and human NMD putative targets showed that most NMD candidates were not orthologous among these species [4]. As a possible alternative explanation for the latter finding is the existence of different PTC-recognition mechanisms in each species [4], here we consider two species with the same PTC-recognition mechanism, namely mouse and human.

Based on RefSeq mRNAs of high quality, we systematically and computationally identify NMD candidates in both species, according to the well-defined mammalian NMD 55-nt rule [16] (Figure 1) and employ this set to attempt to distinguish the noisy splicing from the regulation model. We start by defining the data set, ensuring that PTCs are not rare alleles or sequencing artefacts. We then consider the functional and evolutionary properties of NMD candidates. We find that NMD candidates are not commonly conserved between humans and mice. NMD-specific exons are rich in young and low-inclusion-level exons. Although the NMD-specific exons have a high ratio of non-synonymous (K_a_) to synonymous (K_s_) substitution rate, neutral evolution can be rejected. We find no significant enrichment of NMD candidates in the class of genes subject to positive selection.

## Results

### Two to four percent of RefSeq genes are nonsense-mediated decay candidates and not explained as rare alleles or sequencing artefacts

NMD candidates were identified in human and mouse from the RefSeq mRNA databases [45,46]. The RefSeq database contains many partially manually curated mRNA sequences, especially for those prefixed with 'NM_', which generally have experimental support. Based on these 'NM_'-prefixed mRNAs and following the NMD 55-nt rule [16], we identified 701 and 498 NMD candidate genes in human and mouse, respectively (Table 1). These represent 3.9% and 2.8% of genes examined in human and mouse, respectively. The proportions are at the lower boundary of previous reports [4,9-12]. This may in part reflect the exclusion of many splicing isoforms, owing to a lack of adequate experimental support, in the RefSeq database [47]. On the other hand, previously reported proportions of regulated genes were mostly based on modulation of expression profiles and should reflect both the direct and indirect effects of NMD [31,39,48]. *A priori *such methods are expected to overestimate the number of genuine NMD targets [37].

**Table 1:** Comparison of the gene structures of mouse and human nonsense-mediated mRNA decay and non-nonsense-mediated mRNA decay candidates

	Human			Mouse		
		
	NMD	Non-NMD	*P*	NMD	Non-NMD	*P*
Average intron length	3,001	2,641.2	1.1 × 10^-3^	2,399.1	1,957.1	2.1 × 10^-5^
5' UTR length	145	132	8.4 × 10^-4^	124	102	7.0 × 10^-6^
3' UTR length	1,049	618	2.9 × 10^-28^	1134	513	4.4 × 10^-47^
Protein length	341	420	5.6 × 10^-12^	337	378	1.6 × 10^-6^

Total number of genes	701	17,498		498	17,479	

Notes. NMD = Nonsense-mediated mRNA decay. NMD: NMD candidates; Non-NMD: genes that are not NMD candidates. The median values for each parameter are listed because the mean values were not suitable for highly skewed distributions. The *P *value was determined using the Wilcoxon rank-sum (Mann-Whitney) test.

By reference to the Mammalian Gene Collection full length cDNAs [49,50] and dbSNP [51] data (Table S1 in Additional file 1), we find that the NMD candidates have the same quality support as other genes, indicating that these candidates are not the result of sequence artefacts or rare alleles. We also found that human NMD candidates here are enriched for NMD potential targets, previously determined in the study by Mendell *et al *[10] (Table S2 in Additional file 1). As shown in Table 1, NMD candidates generally encode shorter proteins and longer introns and UTRs compared with non-NMD genes.

### Few genes are nonsense-mediated decay candidates in both mouse and human

The splicing noise model predicts that an NMD gene in one species need not be the target of NMD in another. The regulation model does not necessarily predict this. Based on the ortholog pairs identified with the Inparanoid program [52], we counted the number of ortholog pairs that were NMD candidates in both species (Table 2). Only 24 ortholog pairs (6.70% and 8.22% of human and mouse NMD candidates, respectively) were NMD candidates in both species (see the Additional file 2 for complete list of conserved NMD candidates).

**Table 2:** Few genes are nonsense-mediated mRNA decay candidates both in humans and mice.

	Human	Mouse
Number of orthologs	13,120	13,120
NMD candidates in either species	358	292
NMD candidates in both species	24	24

NMD = nonsense-mediated mRNA decay

As it is likely that some NMD transcripts were missed from the analysis due to strict data selection, we repeated the analysis using all the RefSeq mRNAs, including predicted mRNAs (with prefix 'XM_'). The number of NMD candidates in both genomes increased as expected in this second round. However, the intersection between NMD orthologs was still small (see Table S3 in Additional file 1). The result is consistent with a previous comparison of human, fruitfly *D. melanogaster *and *S. cerevisiae *NMD candidates [4,9-12]. We can also show that low rates of NMD conservation are not consistent with the normal rates of stop-codon turnover (see Table S4 in Additional file 1). Our finding shows that, even when the mechanism of PTC regulation is not a variable, deterministic regulation by NMD is generally not selectively favoured over the long term or is not the correct explanation for most PTCs.

### Nonsense-mediated decay exons tend not to be multiples of three long

If an exon is included by noisy splicing, pressure not to be translated should be higher if the exon is not a multiple of three long than if it is a multiple of three long. The inclusion of such exons will induce a frame shift if translated, which will change the encoded amino acids downstream of included exons. If so, this will result in proteins that are at best costly to make and non-functional, and at worst are toxic to cells. In contrast, inclusion of an exon that is a multiple of three will typically result in a small peptide insert but need not disrupt the overall function of that protein. The costs of noisy splicing can be reduced to some extent by degradation of these noisy transcripts employing mRNA decay systems, such as by NMD. As exons that are not a multiple of three impose a higher cost, we expect stronger selection for PTCs in exons that are not multiples of three compared with those that do not induce frameshifts, assuming an equal rate of mis-splicing. The regulation model makes no prediction of bias.

We classified exons in NMD candidates as NMD-specific exons (the exons observed only in NMD transcripts) and NMD-nonspecific exons (the left exons in NMD candidate genes) (Table 3). We began with genes having at least two RefSeq mRNAs and obtained 36,643 internal coding exons from 3,362 genes (including 252 NMD candidates and 3,110 non-NMD genes) (Table 3). Among these, we identified 278 NMD-specific exons and 2,353 NMD-nonspecific exons. Since NMD-specific exons may tend to be owned by only one transcript, for better comparison we classified exons in non-NMD candidates as non-NMD-single (exons observed only in one RefSeq mRNA) and non-NMD-multiple exons (the remaining exons in non-NMD candidate genes) (Table 3). We focused on the NMD-specific exons of cassette type, because these exons did not overlap with regions in non-NMD transcripts and were more suitable for our purpose.

**Table 3:** Classification of human exon types based on RefSeq data

Exon class	Constitutive	Alternative (Non-cassette)	Alternative (Cassette)^c^	Total
NMD-specific^a^	0	142	136 [82]	278
NMD-non-specific^a^	1,599	282	472 [251]	2,353
NonNMD-single^b^	0	1697	4,110 [2,165]	5,807
NonNMD-multiple^b^	25,236	651	2,318 [1,137]	28,205

Notes. NMD = nonsense-mediated mRNA decay. ^a ^the NMD-specific exons are only observed in NMD transcripts, and the left exons of NMD candidate genes are classified as NMD-nonspecific. ^b ^the NonNMD-single exons are the ones observed only in one transcript, and the left exons of Non-NMD candidates are called as NonNMD-multiple. ^c ^the numbers in the square brackets are the counts of cassette exons whose lengths are not divided exactly by three. Cassette exons are the alternative exons completely skipped or included in splicing isoforms.

As shown in the fourth column of Table 3, the lengths of NMD-specific cassette exons are not divisible by three in more cases (60.3%) than non-NMD cassette exons (52.7% and 49.1% for non-NMD-single and non-NMD-multiple, respectively, chi-square test, *P *values: 0.09616 and 0.01388). This supports the noisy splice model. Note too that human-mouse conserved NMD exons show less of a tendency to not be multiples of three (14 of 23 = 60.8%) than NMD exons created after the mouse-human split (7 of 9 = 78%), although sample sizes are too small to make definitive conclusions (See Table 4 for conserved exons).

**Table 4:** Comparison of exon creation/loss between human nonsense-mediated mRNA decay-specific and non nonsense-mediated mRNA decay-single exons

Evolutionary patterns	Human (source)	Mouse (target)	Dog (outgroup)	NMD-specific	NonNMD-single
Conserved in target	+	+	+	23	436
Conserved in target	+	+	-	1	13
Lost in target	+	-	+	2	38
Created in source	+	-	-	9	66

Total				35	553

Notes. NMD = nonsense-mediated mRNA decay. +, The orthologous exon exists in this lineage; -, The orthologous exon is not observed in this lineage.

### Nonsense-mediated decay-specific exons tend to be in the low inclusion category and newly created

According to the noisy splice model, the NMD transcript is an alternatively spliced unwanted transcript. The regulation model does not require the NMD transcripts to be alternatively spliced transcripts, nor, if they are, need they be the minority form (the transcript isoform that constitutes a small fraction (less than one third) of transcripts from the same gene). Are then NMD genes more likely to be alternatively spliced than random genes and are the NMD transcript isoforms rare? To investigate this we mapped our gene lists to Ensembl genes with BioMart [53], and extracted the splicing isoform information from the ASD database [54,55].

We find that 419 out of 458 and 271 out of 347 (for humans and mice, respectively) NMD candidate genes have at least two known splicing isoforms in ASD [54,55] (Table 5). Generally, one NMD candidate gene may have both NMD and non-NMD transcript variants. Compared with randomly selected genes we find that NMD candidates are more commonly subject to alternative splicing in humans (*P *= 0.0007832). However, there is no difference in mouse (*P *= 0.8705). This is probably caused by: 1) misclassifying some true NMD targets into non-NMD set in mouse due to smaller number of 'NM_' prefixed RefSeq mRNAs (19,083 and 23,839 RefSeq mRNAs in our dataset for mice and humans, respectively); and 2) lower detection rate of alternative splicing for mice (79%) than humans (86%) in the ASD database [54,55]. Note too that Xing and Lee found that in rodents NMD candidates are subject to alternative splicing more commonly than expected [44].

**Table 5:** Identification of alternatively spliced genes based on the ASD database

	Human		Mouse	
		
	AS	Non-AS	AS	Non-AS
NMD	419	39	271	76
Non-NMD	12047	1987	9761	2656

Notes. NMD = nonsense-mediated mRNA decay. In each cell are the counts of genes for each category. AS: the genes have alternative splicing isoforms identified in ASD database [54]. Non-AS: these genes have only one transcript isoform for each. Chi-square test: *P *= 0.0007832 and 0.8705 for human and mouse, respectively.

**Figure 2:**
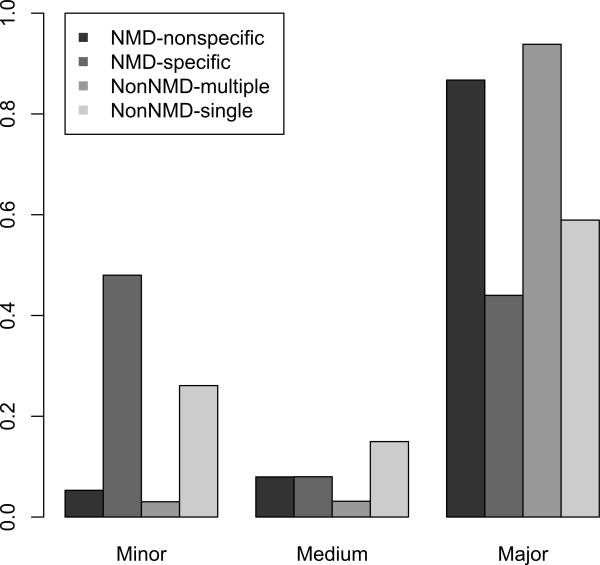
**Exon inclusion levels in different classes of cassette exons**. The nonsense-mediated mRNA decay-specific exons are enriched in the minor-form (inclusion level < 33%) inclusion category and depleted in the major-form (inclusion level > 67%) category.

As expected from the noisy splicing model we found that a larger proportion of NMD-specific exons (48%) were spliced in minor form (included in less than one third of transcripts transcribed from this gene) compared with non-NMD-single exons (26.1%) (Figure 2) (One-sided Kolmogorov-Smirnov test, *P *= 0.02443). This is consistent with previous reports that most putative NMD transcripts are expressed in low abundance across examined tissues [41] and with the finding that both in humans and mice the minor transcript has a PTC more commonly than the major form (11.1% *versus *3.7%) [44].

Are NMD exons ancient or new? To explore this question, we examined the exon creation and loss events for each NMD-specific cassette exon. To obtain this information, we started by mapping our exons to those from the ASAP2 and the VEEDB databases [56,57]. The VEEDB database provides exon conservation information for each given exon based on splice site conservation, this being extracted from a 17-vertebrate UCSC multi-genome alignment [57]. Using this exon conservation data, we could determine whether a given human exon is conserved or absent in mouse and dog (outgroup). Unfortunately, only a small portion of exons are mapped to the VEEDB database [57]. As shown in Table 4, 9 out of 35 NMD-specific exons were created after the human-mouse split. This proportion (25.7%) is significantly higher than that of non-NMD-single class exons (11.9%) (Fisher's exact test, *P *= 0.0315) (see Additional file 2 for human NMD-specific exons conserved in mice). When we compared humans against Rhesus macaques (*Macaca mulatta*) with mice as outgroup, the difference is more significant (Additional file 1, Table S5, NMD-specific 21.2%, non-NMD-single 4.5%, *P *= 0.001139). These findings are consistent with the fact that NMD-inducing exons are often not conserved among species [41]. The exon loss rates are much smaller and there was no difference between NMD and non-NMD exons.

An association between new and alternatively spliced exons can probably also account for the rapid turnover of genes subject to NMD. Two types of species-specific alternative splicing events can be defined [58,59]. One type, referred to as 'species-specific alternative splicing of conserved exons', is represented by a conserved exon that is alternatively spliced in one species but constitutively spliced in the other species. The other type, referred to as 'genome-specific alternative splicing', is represented by an alternative exon in one species which is not detectable in the ortholog of the other species (see Figure Three in [41] for diagrams). More than 41% of species-specific alternative splicing events of conserved exons and 61% genome-specific alternative splicing events had the potential to trigger NMD, while the NMD-inducing rate of conserved alternative splicing events between human and mice was much lower (< 31%) [41]. No matter which form it is, both types cause NMD to occur more often in only one of the two species than in both species, and can hence explain to a considerable extent NMD status divergence.

### Where an orthologous exon can be found, the nonsense-mediated decay-specific exon is or was under purifying selection

Both models (noise and expression regulation) predict that NMD exons may be under lower selective constraint than non-NMD exons. However the noisy model predicts neutral evolution for the NMD-specific exons (owing to their not being translated) while the regulation model predicts there to be purifying selection still operating. To examine the evolution of NMD-specific exons, we concatenated the alignments of the NMD-specific cassette exons based on human-mouse-dog CDS alignments and calculated *K*_a_/*K*_s _ratios (ω) of non-synonymous substitution rate (*K*_a_) to synonymous substitution rate (*K*_s_) for each lineage using the PAML package [60] under the free-ratio branch model [61], which assumes that each lineage evolved independently. As expected (Table 6), the *K*_a_/*K*_s _ratio in human NMD-specific cassette exons is higher than that in other regions of NMD candidates (0.3432 versus 0.2627). This is consistent with weaker negative selection pressure on the NMD exons. It is also higher than the ratios for the orthologous exon in other lineages (0.1082 and 0.0884 for mouse and dog, respectively).

**Table 6:** *K*_a_/*K*_s _ratios comparison between human nonsense-mediated mRNA decay and Non-nonsense-mediated mRNA decay concatenated alignments.

	NMD-specific exon alignment^a^	Remained region in NMD alignment^a^	Non-NMD-single exon alignment^b^	Remained region in Non-NMD alignment^b^
ω (Human)	0.3423	0.2627	0.2554	0.1668
ω(Mouse)	0.1082	0.2184	0.1835	0.128
ω(Dog)	0.0884	0.2509	0.2193	0.1676

Notes. NMD = nonsense-mediated mRNA decay. ω (Human), ω (Mouse) and ω (Dog) are the *K*_a_/*K*_s _ratios in human, mouse and dog lineages, respectively. ^a ^NMD-specific exon alignment was concatenated alignment from the human NMD-specific exon regions of human-mouse-dog three-way CDS alignments, and the rest regions in the CDS alignments were concatenated together to construct the remained NMD alignment. ^b ^similar to the NMD-specific case, the human non-NMD-single exon regions of human-mouse-dog three-way CDS alignments were clipped out to construct the concatenated alignment, and the rest regions to construct the remained non-NMD alignment. See Methods for details.

Comparing alternative cassette exons with other parts of the same gene may be an error-prone test as cassette exons may generally have weaker evolutionary pressures due to their exclusions in some splicing isoforms. Indeed, we observe a higher *K*_a_/*K*_s _ratio in non-NMD-single class of exons than that in other regions (Table 6). To exclude the effect of this, we compared the NMD-specific *K*_a_/*K*_s _ratio against non-NMD-single exons. A higher *K*_a_/*K*_s _ratio for the NMD-specific class was still observed (Table 6, 0.3423 versus 0.2554).

While these results are consistent with the regulated expression model, fuller interpretation of the results is non-trivial. First, the ability to detect orthologous exons predisposes to finding exons functioning in regulation. Second, even if the spurious transcript model is correct for the lineage with the stop, the null for *K*_a_/*K*_s _is not 1. Crucial is when the exon was subject to NMD and what form of selection operated prior to this. If the exon was subject to NMD very recently, then most of the evolutionary history of the exon down the human lineage was not evolving in response to the presence of the stop. Only if the exon was always spurious, unlikely given that this exon is found in multiple distant taxa, would *K*_a_/*K*_s _= 1 be expected for the lineage in question. In short, by virtue of the fact that we can find distant orthologous exons, we are almost certainly biasing the data set to exons that have been and possibly still are functional. Given that *K*_a_/*K*_s _< 1 we can be confident that for some of the time the exon has not been spurious.

### Nonsense-mediated decay candidates are fast evolving but not hot-spots for adaptive evolution

Faster evolution for NMD-specific exons and higher *K*_a_/*K*_s _ratios for NMD candidates (even when controlling the expression profiles) (Figure S1 and Table S6 in Additional file 1) caused us to wonder whether the evolutionary mode of these genes is purifying selection, neutral evolution or adaptive evolution. Since most NMD candidates showed *K*_a_/*K*_s _< 1 (Figure S2 in Additional file 1), these genes were under purifying selection as a whole. Consistent with this, we found that the NMD-specific cassette exons rejected the neutral evolution model (*K*_a_/*K*_s _= 1) using a likelihood ratio test [61] (Table 7, *P *= 1.77 × 10^-8^), although they were fast evolving compared with other regions in NMD candidates (Table 6).

**Table 7:** Test of neutral evolution for human nonsense-mediated mRNA decay-specific exons under branch model.

Model	ω (Human)	ω (Mouse)	ω (Dog)	Log-likelihood	Number of parameters	*P *value
Free-ratios	0.3423	0.1082	0.0884	-6947.6716	7	
Free-ratios(fix)	1	0.0973	0.0732	-6963.5371	6	1.77E-08

Note: if the neutral evolution occurred in human lineage, then the log-likelihood should not change much after the ω is fixed at 1. Our data reject this neutral model with a significant difference.

Given that NMD candidates evolve faster in their own lineage than orthologs (relative rate test, Table S7 in Additional file 1), possibly owing to reduced selective constraints, it is tempting to suppose that NMD genes and exons are potentially given much more freedom to roam sequence space than exons of, for example, house-keeping genes. Might this predispose NMD genes to be hot-spots for adaptive evolution?

We checked if there were any cases of relaxed selection or positive selection for NMD candidates based on data from a previous study [62], which used a sensitive method (branch-site model in PAML [63,64]) to detect positive selection sites in human and chimp genes. As shown in Table 8, of 8,824 genes 104 genes were considered under positive selection in the human lineage. Four of 254 NMD candidates were detected as being candidates for positive selected. This proportion (1.6%) is slightly higher than that (1.2%) of non-NMD genes, but not statistically significant (*P *value = 0.5453). We find no evidence that NMD candidates are under relaxed selection (Table S8 in Additional file 1) for the 254 examined NMD candidates, indicating that, like most other genes, they are under purifying selection.

**Table 8:** The frequency of positive selection in human genes is not correlated with nonsense-mediated mRNA decay status.

	PS	Non-PS	Total
NMD	4	250	254
Non-NMD	100	8470	8570

Note. NMD = nonsense-mediated mRNA decay. The information on positive selection (PS) was downloaded from the supplementary data in study [62]. Fisher's exact test, *P *= 0.5453.

### Under-representation of some functional classes of genes in the nonsense-mediated decay set is consistent with noisy splicing

The above results suggest that for modern exons the noisy splicing model is appropriate. One result in this context is curious. While a gene that is an NMD candidate is unlikely to be an NMD candidate in other species, we do see that some functional classes of genes are consistently under-represented as NMD candidates (Table 9 and Additional file 3) and the proportion of NMD candidates within any given gene ontology (GO) class is largely unaltered between mouse and human (see Additional file 4). At first sight this conservation of function and the skew in representation looks like evidence for regulated splicing which predicts that NMD regulation might be particular for certain types of genes (for example, starvation or hypoxia-specific genes). We, however, note that this skew is also potentially consistent with the noisy splicing model, if there is a covariance between gene classes and rates of alternative splicing.

**Table 9:** Biological process analysis of nonsense-mediated mRNA decay candidates.

	Human			Mouse		
		
Biological process terms	All genes	NMD genes	*P *value	All genes	NMD genes	*P *value
Biological process unclassified	6,172	327^O^	1.45 × 10^-11^	6080	244^O^	1.28 × 10^-11^
Developmental processes	1,908	43^U^	1.68 × 10^-3^	1867	32^U^	5.70 × 10^-2^
Cell-surface receptor-mediated signal transduction	1,513	30^U^	3.56 × 10^-3^	-	-	-
Sensory perception	465	5^U^	1.08 × 10^-2^	971	13^U^	7.10 × 10^-2^
Signal transduction	3,127	87^U^	1.44 × 10^-2^	3491	66^U^	8.75 × 10^-3^
Chemosensory perception	203	0	6.03 × 10^-2^	545	2^U^	5.14 × 10^-3^
Olfaction	-	-	-	539	2^U^	8.14 × 10^-3^

Total number of genes	18,172	693		17939	491	

Notes. NMD = nonsense-mediated mRNA decay. The biological processes with Bonferroni-corrected *P *values < 0.1 are listed; the complete result is presented in Additional file 3. ^O^: biological process was over-represented in NMD candidates; ^U^: biological process was under-represented in NMD candidates; symbol '-' indicates that the biological process exhibited a *P *value > 0.1 in this species, but not in the other species.

To examine the skew we employed tools of PANTHER database [65,66]. The biological processes with Bonferroni-corrected *P *values < 0.1 in either species are listed in Table 9. About half of the NMD candidates were classified as *Biological process unclassified*. This was the only set showing over-representation with the NMD class. The only other classes showing significant deviation from expected showed under-representation, these being *Developmental processes, Cell-surface receptor-mediated signal transduction, Sensory perception, Signal transduction, Chemosensory perception*, and *Olfaction*. The functional distributions of NMD candidates in humans and mice were quite similar. Of the top six most significant GO terms from either species, five (*Biological process unclassified*, *Developmental processes, Sensory perception, Signal transduction, Chemosensory perception*) also appeared in the list of the most significant in the other species (Table 9).

To further determine whether the divergence of orthology in NMD candidates also led to functional divergence, we compared the functional distributions of human and mouse NMD candidates using the FatiGO web tool [67], which is able to detect particular GO terms for which the two lists of genes have different proportions of genes. For feasibility, the GO terms for mouse genes were deduced from the corresponding human orthologs. No significant GO terms were detected at any level (GO levels 3 to 9; see Additional file 4). To exclude the effect of orthologs that are NMD candidates in both species, we repeated the analysis after removing these orthologs from either or both species. As before there was no GO term showing a significant difference between mice and humans in the regularity of NMD (data not shown). This indicates that there is no functional class in which there are significantly more or fewer human NMD candidates compared against mouse NMD candidates.

These results suggest that NMD targets different genes in the two species, but ones largely in the same functional categories (Table 9 and Additional file 4). While superficially this looks like evidence for regulated splicing, if alternatively spliced genes are more prone to incorrect splicing, we expect, under the noisy splicing model, that the NMD-under-represented functional classes will have fewer alternatively spliced genes than other classes.

To test this, we extracted the genes associated with each functional class in Table 9 (excluding the *Biological process unclassified *class) for humans from the PANTHER database [65,66]. Then, we compared the proportion of alternatively spliced genes in each class against the rest. As shown in Table 10, in total 12,466 out of 14,492 (86%) genes are alternatively spliced based on the ASD database [54,55], while the proportions for classes *Developmental processes, Cell-surface receptor-mediated signal transduction, Sensory perception, Signal transduction *and *Chemosensory perception *are significantly smaller. Repeating the same analysis after removing all the NMD genes we find the same result (Table 10). These results suggest that covariation of functional class with NMD is consistent with the noisy splice model and different regularities of alternative splicing.

**Table 10:** Evidence that there are fewer alternatively spliced genes in nonsense-mediated mRNA decay under-represented functional classes in humans.

	All^a^				Non-NMD^b^			
		
Biological process terms	Non-AS	AS	AS Proportion	*P *value	Non-AS	AS	AS proportion	*P *value
Developmental processes	286	1,304	0.82	1.27E-06	283	1271	0.82	1.43E-06
Cell surface receptor mediated signal transduction	224	834	0.79	3.40E-12	221	811	0.79	5.19E-12
Sensory perception	44	170	0.79	0.00699	44	165	0.79	0.00543
Signal transduction	416	2,029	0.83	2.44E-06	409	1960	0.83	2.31E-06
Chemosensory perception	5	5	0.50	0.00726	5	5	0.50	0.00767
Total genes	2,026	12,466	0.86		1987	12,047	0.86	

Notes. NMD = nonsense-mediated mRNA decay. ^a ^all the genes with alternative splicing information were used. ^b ^only the genes that are not NMD candidates were used.

## Discussion

While NMD must play a role in preventing the translation of rare alleles with premature stop codons [23], it is perhaps surprising that 2 to 4% of our genes have a premature stop codon that is not just a rare allele (Table S1 in Additional file 1). While it is likely that in some cases (ancient exons) NMD functions in a regulatory mode, our results more strongly support the noisy splicing model. Many features are consistent with this: the rarity of genes regulated by NMD in one species being regulated by NMD in the other (controlling for PTC recognition mechanism) (Table 2 and Table S3 in Additional file 1); the excess of exons that are not multiples of three long (Table 3); the association with alternative splicing (Table 5) and with minor splice forms (Figure 2); and the excess of modern exons associated with NMD (Table 4 and Table S5 in Additional file 1). Conservation of the gene classes subject to NMD (Table 9 and Additional file 4) is also consistent, given that gene class and propensity for alternative splicing covary (Table 10). These results are consistent with previous studies and extend their findings.

Is our estimate of 2 to 4% of genes being subject to NMD accurate? This estimate is on the lower bounds, compared with prior approaches [9-12]. This likely reflects in part both our conservative approach and a tendency for alternative methods to over-estimate. As regards the latter, previous studies based on EST data [40,42,43] or expression microarrays [10] found higher proportions of NMD candidates. However this may include some aberrant transcripts due to noise in EST data [68]. More problematic is the possibility that, as candidates are identified based on expression profile changes after inhibition of NMD, many indirect NMD targets are included [31,39] (for example, those up-regulated by a protein made from an NMD-regulated gene). There are, however, at least two reasons why our study might be conservative. First, because the RefSeq database excludes splice forms without enough experimental support, many true NMD targets may well be missed. Further, in employing the NMD 55-nt rule (Figure 1) to identify the NMD candidates, we may well miss transcripts regulated in a different manner. Notably both extended 3'UTR and uORFs can trigger NMD to some extent [18,19]. Parenthetically, our identified NMD candidates show longer 3'UTRs than non-NMD genes (Table 1). Given an association between long 3'UTR and NMD, it is possible that both long 3'UTRs and an exon junction complex downstream of a PTC contribute to targeting. On balance then our 2 to 4% figure is probably conservative. By equal measure, our NMD sample should be relatively clean (that is, low false positive rate). For this reason we suggest that the results that we present are likely to be robust.

Is it likely that spurious splicing will explain most PTCs in other organisms? Consider, for example, *S. cerevisiae*. Here only 5% of genes have introns and alternative splice forms seem relatively rare. *A priori *in such a genome regulated expression is expected to be the dominant explanation. Nonetheless, a noisy splicing model of some form may yet be viable. In the yeast genome, more than 70% of genomic regions are transcribed [69,70] and the richness of the transcriptome is greater than expected. It is viable to suppose that some fraction of these transcripts is spurious and selection for PTCs, out of the normal reading frame, is selected for. Less clear is how such a model might explain an in-frame PTC in a protein coding gene where the PTC is the unique stop codon in the gene.

## Conclusion

We find good evidence consistent with the noisy splicing model, especially in the case of recent exons. However, for ancient exons with a PTC association with NMD regulated regulation is a viable model.

## Methods

### Data collection

We downloaded the sequences and annotations for human and mice from the NCBI RefSeq [46] database (Build 36.1) in January 2007. To improve the confidence of NMD candidate identification, we only extracted the transcripts with initial letters 'NM_'. Based on these annotations, for each transcript, we calculated the distance from the stop codon to the exon-exon junction closest to the 3' end. According to the NMD rule [3,4,16], we classified transcripts as NMD candidates if the distance was > 55 nucleotide bps. Then, we defined a gene as an NMD candidate if it had at least one NMD candidate transcript. All the remaining genes in the genome were classified as non-NMD candidates.

The UTR length, average intron length and protein length for each transcript were also calculated or extracted from the annotations. For each gene with multiple transcript variants, we collapsed these parameters into one by choosing the splicing form with the longest protein and calculating the means of transcript isoforms.

### Exon type classification

We started from 3,362 genes with at least two RefSeq mRNAs in our dataset, which included 252 NMD and 3,110 Non-NMD candidates. We only considered the coding exons in each transcript and excluded the two marginal 5' and 3' exons within each gene due to their general incompleteness. For NMD candidates, we searched the exon isoforms only observed in NMD transcripts and defined these exons as NMD-specific exons, and defined the remaining exons in NMD candidates as NMD-non-specific exons. For non-NMD candidates, we classified exons as non-NMD-single and non-NMD-multiple. The former were observed in only one splicing transcript isoform for a given gene and the latter were observed in at least two different splicing transcript isoforms.

### Mapping exons to ASAP2 database

Since the ASAP2 database gives the positions of exons on human chromosomes [71] of NCBI build 35.1 [46,72,73], equivalent to UCSC hg17 [74-76], we mapped the human exon set of RefSeq mRNAs to the ASAP2 database [56] as follows: first, we converted the exon positions on reference sequence contigs into those on chromosomes (NCBI build 36.1, UCSC hg18) using a Perl script. Second, we used the UCSC [76] liftOver tool to convert these positions into those on human NCBI build 35.1 (UCSC hg17). Finally, we compared these positions with those in the ASAP2 database and retrieved the ASAP2 exons that exactly matched the RefSeq exon set. 95,029 of 209,222 RefSeq exons can be uniquely mapped to the ASAP2 database. Based on these matched exons, we can easily obtain the splicing state, inclusion levels, exon creation/loss from the ASAP2 and the VEEDB database tables [56,57].

### Construction of exon alignments for nonsense-mediated decay -specific exons and calculation of *K*_a_/*K*_s_

Given the NMD-specific cassette exon lists above, we extracted the corresponding regions from human-mouse-dog CDS alignments (built by a Clustal W, version 1.83 [77], see Additional file 1 for details) and concatenated them together. The remained regions in these alignments were also concatenated. These concatenations were inputted separately into PAML [60] package to calculate *K*_a_/*K*_s _ratio for each lineage under the free ratio model [61]. The alignments for non-NMD-single exons and remaining parts were similarly extracted and inputted into PAML for K_a_/K_s _calculations.

To see if the NMD-specific exons were under neutral evolution, we fixed the *K*_a_/*K*_s _at one in the human NMD lineage under free ratio model [61], and compared this with a more general model (with *K*_a_/*K*_s _free) to test if the neutral model could be rejected using likelihood ratio tests (calculated in R [78]).

### Gene ontology analysis

Comparisons of functional distributions of NMD candidates between human and mouse were carried out using the FatiGO program [67]. FatiGO implements the nested inclusive analysis, in which the test is done recursively until the deepest level in which significance is obtained and only this last level is reported. In this way both variables, the efficiency of the test and the highest precision in the term found, are optimized. The program computes a Fisher's two-tail exact test to statistically define over- or under-represented terms between two lists of genes, and the original *P *values are corrected by a false discovery rate approach [79].

The detection of over- or under- represented functional entries for NMD candidates was done based on the PANTHER database [65,66]. The NMD candidate list was compared with the all the genes used and the *P *values were determined using a binomial test for each functional category. The original *P *values were adjusted using a modified Bonferroni correction method, which accounted for the nesting relationship among GO terms at different levels to avoid too conservative corrections.

## Abbreviations

EST: expressed sequence tag; GO: gene ontology; K_a_: non-synonymous substitution rate; K_s_: synonymous substitution rate; NMD: nonsense-mediated mRNA decay; PABP: poly(A)-binding protein; PTC: premature termination codon; uORF: upstream open reading frame; NMD-specific exon: exon observed only in NMD RefSeq transcripts; NMD-non-specific exon: exon in NMD candidate gene but not NMD-specific one; Non-NMD-single exon: exon observed in only one RefSeq transcript in non-NMD candidate genes; Non-NMD-multiple exon: exon shared by at least two RefSeq transcripts in each non-NMD candidate gene; Cassette exon: exon completely alternatively spliced.

## Authors' contributions

ZZ, XK, LDH and conceived and designed the experiments. ZZ performed the computational analysis. ZZ, XK, LH, and LDH analyzed the data. ZZ, DX, PW and LZ contributed reagents/materials/analysis tools. ZZ, LH, XK and LDH wrote the paper. All authors read and approved the final manuscript.

## Supplementary Material

Additional file 1 **Supplementary materials**. This file includes supplementary tables (Table S1–S8), supplementary figures (Figure S1–S2) and methods. Click here for file

Additional file 2 **Conserved nonsense-mediated mRNA decay (NMD) candidates between humans and mice and conserved human NMD-specific exons**. This file contains the list of ortholog pairs which are NMD candidates both in human and mouse, and includes the NMD-specific exons conserved in mice. Click here for file

Additional file 3 **PANTHER analysis of nonsense-mediated mRNA decay candidates in humans and mice**. This file contains the complete results of test of functional biases for human and mouse nonsense-mediated mRNA decay candidates using PANTHER annotating system. Click here for file

Additional file 4 **Comparison of human and mouse nonsense-mediated mRNA decay candidates function distributions**. This file contains the complete results of comparing the functional distributions between human and mouse nonsense-mediated mRNA decay candidates using FatiGO tool. Click here for file
