# Supplementary material for: Noisy splicing, more than expression regulation, explains why some exons are subject to nonsense-mediated mRNA decay
Source: BMC Biol. 2009 May 14;7:23. doi: 10.1186/1741-7007-7-23 (PMC2697156; doi:10.1186/1741-7007-7-23)
Supplement: Additional file 1 — Supplementary materials. This file includes supplementary tables (Table S1–S8), supplementary figures (Figure S1–S2) and methods. [file 1741-7007-7-23-S1.doc]

# Supplementary materials

# Methods

## MGC full length cDNA sequences and SNP data

We downloaded human and mouse full length cDNA sequences from the mammalian gene collection project (http://mgc.nci.nih.gov/) [1]. This dataset contains 26,554 and 24,228 full length cDNA sequences for human and mouse, respectively. For each gene, we mapped all the cDNAs associated with it to all the RefSeq transcripts belonging to it using NCBI MegaBLAST [2, 3]. We selected the best high scoring pair (HSP) for each hit, and examined the coverage of the cDNA and the alignment identity. We removed the HSPs that did not cover the complete cDNA sequences or had identities less than 95%. The full length cDNAs that mapped to more than one RefSeq transcript were also eliminated. Then we counted the number of cDNAs uniquely mapped to each RefSeq transcript and this cDNA should uniquely represent this RefSeq transcript to the maximal extent. For human RefSeq transcript, we also checked whether a SNP existed at the stop codon by extracting SNP information from file ftp://ftp.ncbi.nih.gov/refseq/H_sapiens/mRNA_Prot/ human.rna.gbff.gz, which contains SNP information for each RefSeq transcript based on dbSNP data [4, 5].

## Cross-reference human NMD candidates to the up-regulated genes in [6]

Before cross-referencing our NMD gene set with those up-regulated genes in previous study [6], we filtered some genes as follows: 1) we kept the probesets with which only one gene was associated for each, 2) we extracted probesets with present calls on at least one microarray chip among GSM29530, GSM29531, GSM29532 and GSM29534. This left 5060 probesets, 3) we collapsed the probesets pointing to same genes, resulting in 4113 genes with expression values. We cross-referenced these 4113 genes with our 18200 genes and finally identified 4022 genes appearing in both gene sets, which included 96 NMD candidates.

## Detection of stop codon in mice for NMD stop codons in humans

To see if the large turnover of NMD candidates between humans and mice are caused by mutations at the stop codons, we checked whether the mouse orthologous stop codon exists for each human NMD stop codon. We did this using two types of alignments. First, we aligned the human and mouse orthologous exons containing stop codons (stop-exons) at the nucleotide level using needle (dynamic programming algorithm) program in EMBOSS package [7]. To improve accuracy, we filtered out the alignments in which there were no bases (only gaps) for mouse in the 21-base sub-alignment around the human stop codon positions (extend 9 bps to both sides). These removed alignments could not provide accurate information on how stop codons were changed. In the 211 alignments remaining, we checked whether the stop codon sequences (TAA/TAG/TGA) existed in mouse at the aligned human stop-codon positions. Second, as a supplement we did this using the CDS alignments, which considered the protein-coding information. In detail we divided each NMD stop-exon in humans into the 5’ part and 3’ part relative to the stop codon. Then we align the 5’ part with the coding region of the mouse orthologous exon at the peptide level using needle [7]. To minimize the sample variance in the analysis, we filtered out any alignments if the coding region in either exon was less than 5 bps. We also removed the orthologous exon pairs that are not in the same coding phase. If a functional mouse stop codon exists in an orthologous position to the human stop codon this protocol should identify them. In the remaining alignments, we checked if the stop codons existed in mouse at human NMD stop codons positions. Since we only want to determine the contribution of stop codon mutations to NMD status changes, in both approaches, the alignments in which the mouse ortholog were NMD candidates were removed.

.

**Ka and Ks between orthologous genes**

To see whether NMD candidate genes have different evolutionary rates compared with other genes, we calculated the Ka and Ks for all the orthologs. We downloaded the protein and RNA sequences from the NCBI ftp site (ftp://ftp.ncbi.nih.gov/genomes/) for human, chimpanzee, Rhesus macaques (*Macaca mulatta*), mouse, rat and dog (on 2 January, 2007). For each gene, we chose the longest protein and corresponding mRNA for the subsequent ortholog searches, and Ka and Ks calculations.

Using the Inparanoid program [8], we searched the orthologs for human–chimpanzee, mouse–rat, and human–mouse species pairs. Inparanoid was run with default parameters except for the sequence overlap cutoff, which was set to 0.8 (default is 0.5) for increased specificity. We only kept one-to-one orthologs for the following analysis.

Based on the identified one-to-one orthologous relationships, we first aligned each pair of orthologous proteins with CLUSTALW [9] using default parameters. The corresponding coding sequences (CDSs) were then aligned in-frame with RevTrans [10] using the amino acid alignments as a guide. Finally, the nucleotide alignment was inputted into PAML program to calculate Ka and Ks [11], accounting for codon usage bias and transition:transversion ratio. The datasets are available on request.

**Three-way CDS alignments**

To construct the CDS alignments for human–chimpanzee–macaques orthologs, we extracted the orthologous human–chimpanzee–macaques trios based on the orthologs identified using Inparanoid [8]. We kept only the trios for which each species was represented by just one gene. Proteins of each trio were aligned with CLUSTALW [9] and then mRNA’s coding sequences were aligned using RevTrans [10] as described above.

For mouse-rat-human and human-mouse-dog CDS alignments, we got the ortholog information from HomoloGene database [12]. We downloaded the ‘homologene.data’ file from the NCBI ftp site (ftp://ftp.ncbi.nih.gov/pub/HomoloGene/), and extracted the clusters in which only one gene appeared for each species examined. Based on these 3-way ortholog information, proteins were aligned with CLUSTALW [9] and CDS alignments were got with RevTrans [10].

These alignment datasets are available on request.

**Relative rate test**

To find faster evolved orthologs in human or chimpanzee lineage, we used the human-chimpanzee-macaques CDS alignments (obtained above) as input to RRTree [13] to do relative rate tests. The *p* values were corrected for multiple testing using the method of Benjamini and Hochberg [14] implemented in R package [15].

The test between mouse and rat was also performed in the same way with the mouse-rat-human 3-way CDS alignments as input to RRTree [13].

The datasets are available on request.

**Expression analysis**

To determine the expression level for each gene used in our analysis, we downloaded expression data from the Genomics Institute of the Novartis Research Foundation (GNF) [16] (<http://wombat.gnf.org/index.html>), which includes 79 human tissues and 61 mouse tissues. Then we calculated the maximum expression (MaxExp), median expression (MedianExp) and expression breadth (Breadth) across the tissues examined. The expression breadth is the number of tissues in which the gene is expressed in terms of A(absent)/P(present) call values of microarray MAS5 algorithm

**References**

1. Strausberg RL, Feingold EA, Klausner RD, Collins FS: **The mammalian gene collection**. *Science* 1999, **286**(5439):455-457.

2. Tatusova TA, Madden TL: **BLAST 2 SEQUENCES, a new tool for comparing protein and nucleotide sequences**. *Fems Microbiology Letters* 1999, **174**(2):247-250.

3. Zhang Z, Schwartz S, Wagner L, Miller W: **A greedy algorithm for aligning DNA sequences**. *Journal of Computational Biology* 2000, **7**(1-2):203-214.

4. Smigielski EM, Sirotkin K, Ward M, Sherry ST: **dbSNP: a database of single nucleotide polymorphisms**. *Nucleic Acids Research* 2000, **28**(1):352-355.

5. Sherry ST, Ward MH, Kholodov M, Baker J, Phan L, Smigielski EM, Sirotkin K: **dbSNP: the NCBI database of genetic variation**. *Nucleic Acids Research* 2001, **29**(1):308-311.

6. Mendell JT, Sharifi NA, Meyers JL, Martinez-Murillo F, Dietz HC: **Nonsense surveillance regulates expression of diverse classes of mammalian transcripts and mutes genomic noise**. *Nat Genet* 2004, **36**(10):1073-1078.

7. Rice P, Longden I, Bleasby A: **EMBOSS: the European Molecular Biology Open Software Suite**. *Trends Genet* 2000, **16**(6):276-277.

8. Remm M, Storm CE, Sonnhammer EL: **Automatic clustering of orthologs and in-paralogs from pairwise species comparisons**. *J Mol Biol* 2001, **314**(5):1041-1052.

9. Thompson JD, Higgins DG, Gibson TJ: **CLUSTAL W: improving the sensitivity of progressive multiple sequence alignment through sequence weighting, position-specific gap penalties and weight matrix choice**. *Nucleic Acids Res* 1994, **22**(22):4673-4680.

10. Wernersson R, Pedersen AG: **RevTrans: Multiple alignment of coding DNA from aligned amino acid sequences**. *Nucleic Acids Res* 2003, **31**(13):3537-3539.

11. Yang Z, Nielsen R: **Estimating synonymous and nonsynonymous substitution rates under realistic evolutionary models**. *Mol Biol Evol* 2000, **17**(1):32-43.

12. Wheeler DL, Barrett T, Benson DA, Bryant SH, Canese K, Chetvernin V, Church DM, Dicuccio M, Edgar R, Federhen S *et al*: **Database resources of the National Center for Biotechnology Information**. *Nucleic Acids Res* 2007.

13. Robinson-Rechavi M, Huchon D: **RRTree: relative-rate tests between groups of sequences on a phylogenetic tree**. *Bioinformatics* 2000, **16**(3):296-297.

14. Benjamini Y, Hochberg Y: **Controlling the false discovery rate: A practical and powerful approach to multiple testing**. *J Roy Stat Soc B* 1995, **57**:289-300.

15. Ihaka R, Gentleman R: **R: A Language for Data Analysis and Graphics**. *J Comput Graph Stat* 1996, **5**(3):299-314.

16. Su AI, Wiltshire T, Batalov S, Lapp H, Ching KA, Block D, Zhang J, Soden R, Hayakawa M, Kreiman G *et al*: **A gene atlas of the mouse and human protein-encoding transcriptomes**. *Proc Natl Acad Sci U S A* 2004, **101**(16):6062-6067.

17. Miller W, Rosenbloom K, Hardison RC, Hou M, Taylor J, Raney B, Burhans R, King DC, Baertsch R, Blankenberg D *et al*: **28-way vertebrate alignment and conservation track in the UCSC Genome Browser**. *Genome Res* 2007, **17**(12):1797-1808.

18. Arbiza L, Dopazo J, Dopazo H: **Positive selection, relaxation, and acceleration in the evolution of the human and chimp genome**. *PLoS Comput Biol* 2006, **2**(4):e38.

# Figures


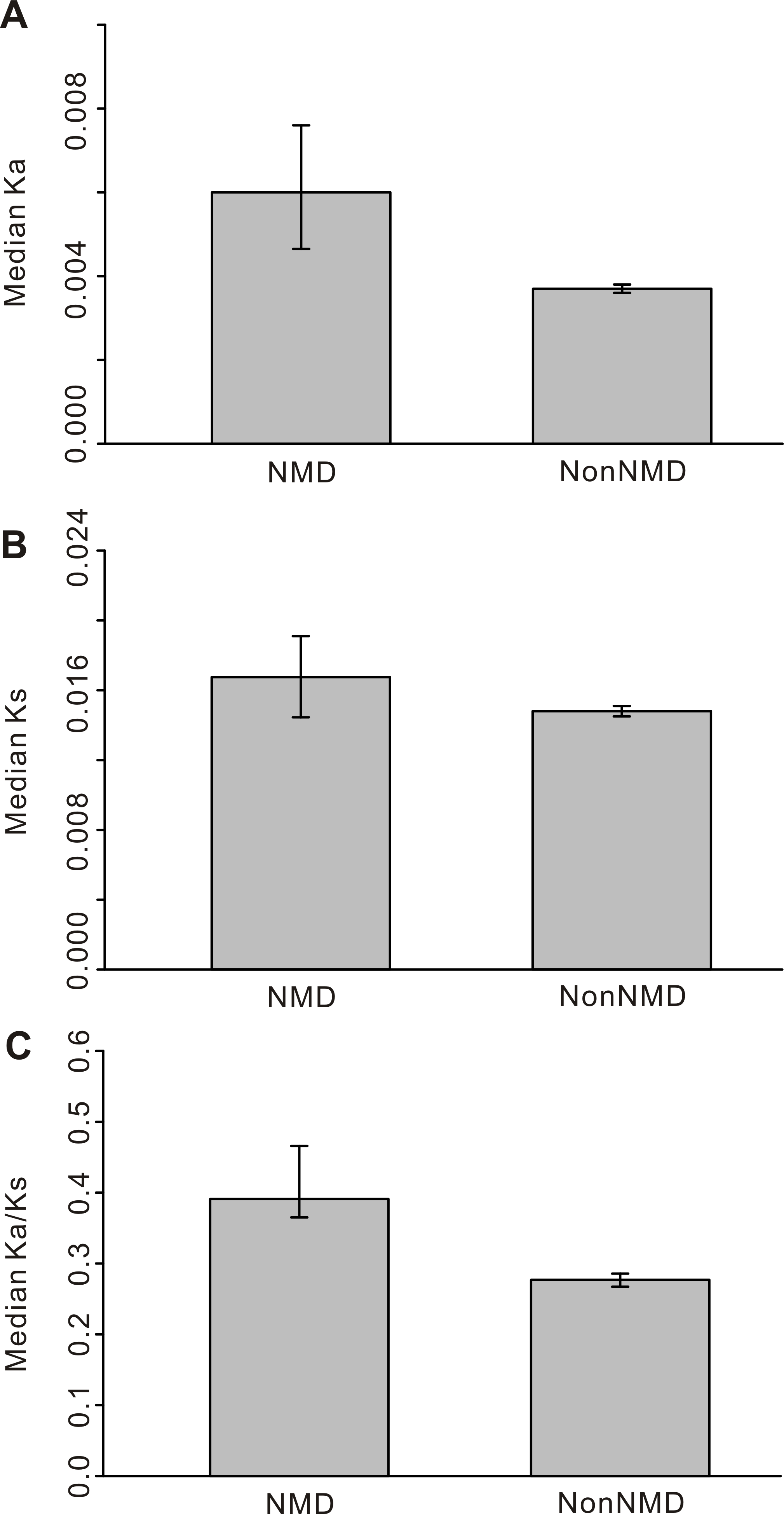


## Figure S1. NMD targets show higher non-synonymous substitution rates than non-NMD targets. The non-synonymous substitution rates (Ka) (A), synonymous substitution rates (Ks) (B) and the Ka/Ks ratio (C) of human NMD targets and non-NMD genes are compared. For robustness, the medians are shown. Error bars are 95% confidence intervals for group medians based on 10000 bootstrap replicates. The *p* values of Wilcoxon rank-sum test for Ka, Ks and Ka/Ks are 1.68 × 10^-8^, 0.0082 and 1.84 × 10^-8^, respectively.


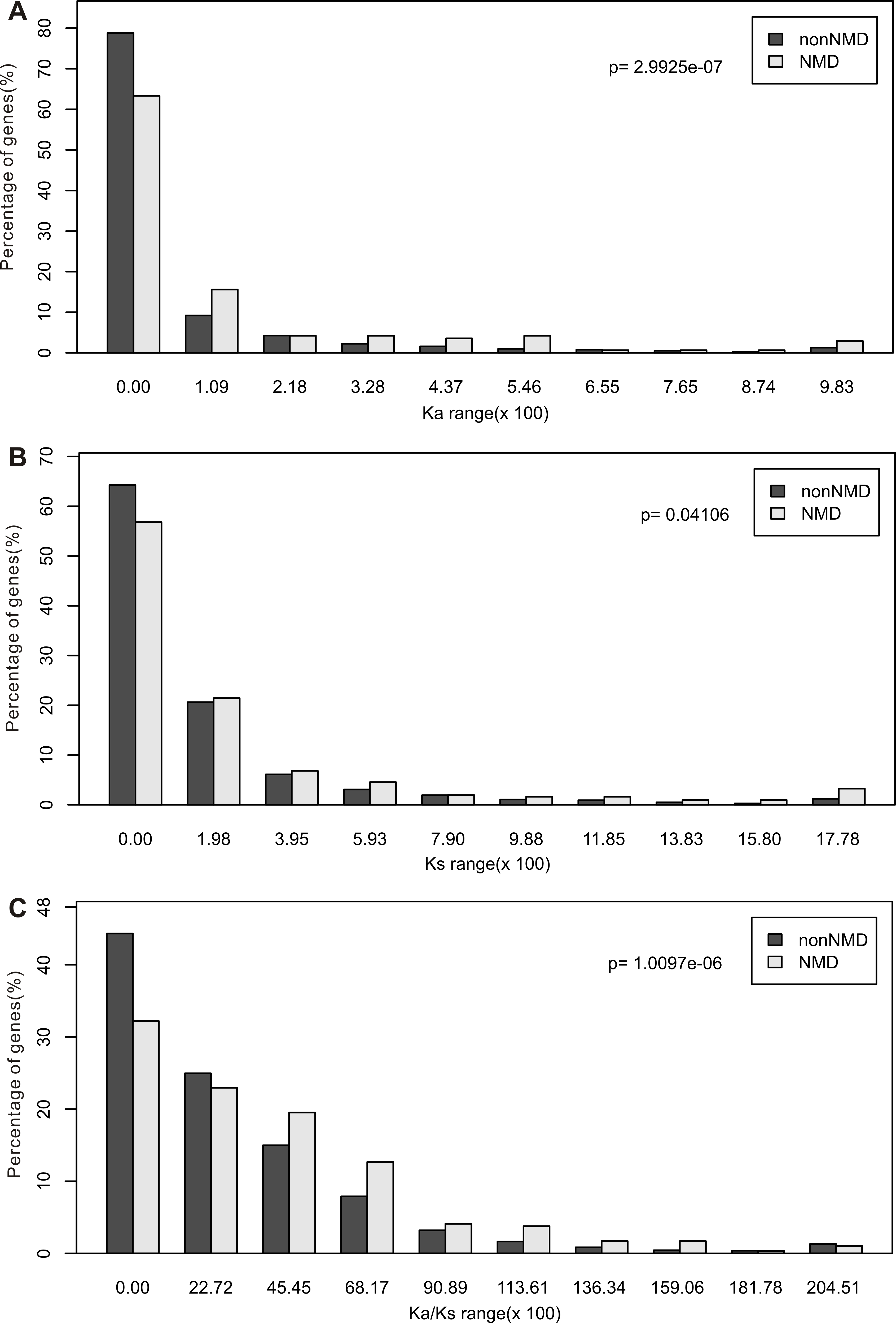


## Figure S2. Distribution comparison of evolutionary rates for human between NMD and Non-NMD candidates. (A) Ka, (B) Ks, and (C) Ka/Ks. The numerical values on the x-axis are the starting values for each group. The original Ka, Ks and Ka/Ks are multiplied by 100 for better presentation.

# Tables

Table S1. Evaluations of the sequence qualities by reference to MGC full length cDNAs and SNP data.

A. Human

|  | Supported^a^ | Unsupported^a^ | Proportion |
| --- | --- | --- | --- |
| NMD | 144 | 519 | 0.217195 |
| NonNMD | 6896 | 14171 | 0.327337 |

B. Mouse

|  | Supported^a^ | Unsupported^a^ | Proportion |
| --- | --- | --- | --- |
| NMD | 158 | 246 | 0.391089 |
| NonNMD | 6462 | 10226 | 0.387224 |

Notes. a: supported denotes for a RefSeq mRNA a full length cDNA is specifically and completely mapped on it and the identity of alignment region larger than 95%. Unsupported means for this mRNA no specifically mapped full length cDNA is found.

**C.** Human SNPs at the stop codons

|  | With_SNP | No_SNP |
| --- | --- | --- |
| NMD | 3 | 121 |
| NonNMD | 151 | 16890 |

Notes. The number in each cell is counts of stop codons. With_SNP means that a SNP is found in the stop codon, and No_SNP means that no SNP is found in this stop codon. 2 of the three NMD SNPs (rs10263734 and rs6661174) changes stop codons to amino acids. Furthermore, rs6661174 (alleles C/T) has frequency data, and the T allele frequencies in population HapMap-CEU, HapMap-HCB, HapMap-JPT and HapMap-YRI are 1.0, 1.0, 1.0 and 0.825 respectively. This snp is at the first position of stop codon of transcript NM_001460.2, so T allele produces stop codon. If the NMD transcripts are rare alleles, we should easily find SNPs at the stop codons and the major alleles should produce amino acids, but not termination codons. In fact, our observations contrast with this inference. Therefore, the NMD transcripts should not be the result of rare alleles

**Table S2** Human NMD candidates are abundant in up-regulated genes when inhibiting NMD

|  | Expressed Genes | Upregulated Genes | Upregulated Genes(known NMD^a^) |
| --- | --- | --- | --- |
| Intersect with all gene set | 4022 | 159 | 77 |
| Intersect with NMD candidate set | 96 | 10 | 8 |
| Fisher exact p value |  | 0.004217 | 0.000427 |
| odds ratio |  | 2.946261 | 5.077198 |

Notes. a: ‘known NMD’ means there is a known NMD mechanism given for this gene in Table S1 in [6]. See supplementary Methods for details.

**Table S3**. The small intersection of NMD candidates using all the RefSeq mRNAs

|  | Human | Mouse |
| --- | --- | --- |
| Number of orthologous genes | 14932 | 14932 |
| NMD candidates | 421 | 343 |
| NMD candidates in both species | 29 | 29 |

**Table S4.** Low rates of NMD conservation can not be accounted for by stop codon turnover. 168 out of 211 (79.6%) human NMD stop codons were aligned with mouse triplet bases (TAG/TAA/TGA). In the supplementary analysis using CDS alignments, 70% of NMD candidates of humans had alignable functional stop codons (not PTCs) in mice. These proportions are smaller than the genome average (85.5%=96.8% x 88.3%) based on Miller et al report (Miller et al report that the stop codons of 96.8% human RefSeq genes aligned in mice, of which 88.3% were functional stop codons in mice, based on UCSC 28-way vertebrate alignment (generated using multiz and other tools by aligning 28 vertebrate species, including reptile, amphibian, bird, and fish clades, as well as marsupial, monotreme (platypus), and placental mammals. For details see the conservation track at UCSC http://genome.ucsc.edu/) [17]). However, this decrease could not account for the minimal intersection of NMD candidates between human and mouse (6.7% for human).

|  | Nucleotide-alignment^a^ | CDS-alignment^a^ |
| --- | --- | --- |
| All human NMD stop codons | 211 | 161 |
| With aligned stop codon in mouse | 168 | 113 |

Notes. In each cell are the stop-codon counts under different criteria. ^a^: different types of alignments used to search stop codons in mice. Nucleotide-alignment was produced directly by aligning orthologous exons, while CDS-alignment was created with peptide alignment as guides. See supplementary Methods for details.

**Table S5.** Comparison of exon creation/loss between human NMD-specific and non-NMD-single exons

| Evolutionary patterns | Human | Macaques | Mouse | NMD-specific | NonNMD-single |
| --- | --- | --- | --- | --- | --- |
|  | (source) | (target) | (outgroup) |  |  |
| Conserved in target | + | + | + | 19 | 437 |
| Conserved in target | + | + | - | 7 | 81 |
| Lost in target | + | - | + | 0 | 11 |
| Created in source | + | - | - | 7 | 25 |
| Total |  |  |  | 33 | 554 |

Notes. +, the orthologous exon exists in that lineage; -, the orthologous exon is not observed in that lineage.

**Table S6.** Partial rank correlation analysis of NMD and evolutionary rates

A. Human

| Controlled variables | Ka | |  | Ks | |  | Ka/Ks | |
| --- | --- | --- | --- | --- | --- | --- | --- | --- |
|  | Rho | *P* value |  | Rho | *P* value |  | Rho | *P* value |
| Breadth | 0.0424 | 0.001 |  | 0.0228 | 0.029 |  | 0.0474 | 0.001 |
| MaxExp | 0.0435 | 0.001 |  | 0.0255 | 0.015 |  | 0.0471 | 0.001 |
| MedianExp | 0.0452 | 0.001 |  | 0.025 | 0.017 |  | 0.0488 | 0.001 |
| MaxExp, breadth | 0.0421 | 0.001 |  | 0.0241 | 0.021 |  | 0.0463 | 0.001 |
| MedianExp, breadth | 0.0426 | 0.001 |  | 0.0227 | 0.029 |  | 0.0475 | 0.001 |

B. Mouse

| Controlled variables | Ka | |  | Ks | |  | Ka/Ks | |
| --- | --- | --- | --- | --- | --- | --- | --- | --- |
|  | Rho | *P* value |  | Rho | *P* value |  | Rho | *P* value |
| Breadth | 0.0318 | 0.001 |  | 0.0079 | 0.389 |  | 0.0293 | 0.001 |
| MaxExp | 0.0338 | 0.001 |  | 0.0093 | 0.31 |  | 0.0311 | 0.001 |
| MedianExp | 0.0334 | 0.001 |  | 0.0082 | 0.373 |  | 0.0308 | 0.001 |
| MaxExp, breadth | 0.0318 | 0.001 |  | 0.0084 | 0.359 |  | 0.0291 | 0.002 |
| MedianExp, breadth | 0.0321 | 0.001 |  | 0.0079 | 0.39 |  | 0.0295 | 0.001 |

Notes. Rho: the partial rank correlation coefficient of NMD and Ka, Ks or Ka/Ks when the specific variables are controlled. Here, the variable NMD is 1 if the gene is an NMD candidate, 0 for non-NMD genes. MaxExp: the maximum expression value across the examined tissues. MedianExp: the median expression value. Breadth: the number of tissues in which the gene is expressed. Ka: non-synonymous substitution rate per non-synonymous site. Ks: synonymous substitution rate per synonymous site.

**Table S7.** Rapid evolution for NMD candidates in humans (A) and mice (B)

A. Humans

|  | NMD | |  | Non-NMD | |  |  |
| --- | --- | --- | --- | --- | --- | --- | --- |
|  | >0 | ≤0 |  | >0 | ≤0 | Ratio | *P* |
| Ka(H) - Ka(C) | 101 | 105 |  | 2927 | 4418 | 1.23 | 9.91 × 10^-3^ |
| Ks(H) - Ks(C) | 105 | 101 |  | 3235 | 4113 | 1.16 | 5.63 × 10^-2^ |
| Ka(H)/Ks(H) -Ka(C)/Ks(C) | 106 | 98 |  | 3202 | 4048 | 1.18 | 3.25 × 10^-2^ |
| Ka(H) - Ka(C)^a^ | 16 | 190 |  | 190 | 7155 | 3.00 | 1.83 × 10^-5^ |
| Ks(H) - Ks(C)^a^ | 5 | 201 |  | 97 | 7251 | 1.84 | 2.04 × 10^-1 b^ |

Notes. ^a^: In these rows, it requires a corrected *p* value of < 0.05. ^b^: Fisher exact *p* value was calculated because the expected values were < 5 (Other *p* values were calculated using the Pearson's Chi-squared test.). Ratio is calculated as [column 2/(column2 + column3)]/[column4/(column4 + column5)]. Ka(H) and Ks(H) are the non-synonymous and synonymous substitution rates in the human lineage relative to the out-group Rhesus macaques. Ka(C) and Ks(C) are the non-synonymous and synonymous substitution rates in the chimpanzee lineage relative to the out-group Rhesus macaques.

B. Mice

|  | NMD | |  | Non-NMD | |  |  |
| --- | --- | --- | --- | --- | --- | --- | --- |
|  | >0 | ≤0 |  | >0 | ≤0 | Ratio | *P* |
| Ka(M) - Ka(R) | 167 | 121 |  | 5701 | 6402 | 1.23 | 3.24 × 10^-4^ |
| Ks(M) - Ks(R) | 148 | 140 |  | 5359 | 6800 | 1.17 | 1.86 × 10^-2^ |
| Ka(M)/Ks(M)- Ka(R)/Ks(R) | 181 | 103 |  | 6204 | 5869 | 1.24 | 5.01 × 10^-5^ |
| Ka(M) - Ka(R)^a^ | 35 | 253 |  | 377 | 11726 | 3.90 | 1.15 ×10^-16^ |
| Ks(M) - Ks(R)^a^ | 3 | 285 |  | 30 | 12165 | 4.23 | 3.98 ×10^-2 b^ |

Notes, ^a^: In these rows, it requires a corrected *p* value of < 0.05. ^b^: The fisher exact *p* value was calculated for expected values < 5, other *p* values are Pearson's Chi-squared test**.** Ratio is calculated as [column 2/(column2 + column3)]/[column4/(column4 + column5)]. Ka(M) and Ks(M) are the non-synonymous and synonymous substitution rates in the mouse lineage relative to out-group human. Ka(R) and Ks(R) are the non-synonymous and synonymous substitution rates in the rat lineage relative to out-group human.

**Table S8. Test of relaxed selections for human NMD candidates.**

|  | RSC | Non-RSC | Total |
| --- | --- | --- | --- |
| NMD | 0 | 254 | 254 |
| Non-NMD | 112 | 8458 | 8570 |

Notes. The gene counts of relaxed selection constraint (RSC) and no detection (Non-RSC) are listed in each cell. The information on RSC was downloaded from the supplementary data in study[18].
